# Effect of Divalent Cations and Other Ions on the Tetrahydrofuran Crystal Inhibition of Quaternary Ammonium Salts—Relevance to the Efficiency of Gas Hydrate Quaternary Anti-agglomerants

**DOI:** 10.1021/acsomega.3c02487

**Published:** 2023-06-29

**Authors:** Malcolm A. Kelland, Kjetil Walter Rønning

**Affiliations:** Department of Chemistry, Bioscience and Environmental Engineering, Faculty of Science and Technology, University of Stavanger, N-4036 Stavanger, Norway

## Abstract

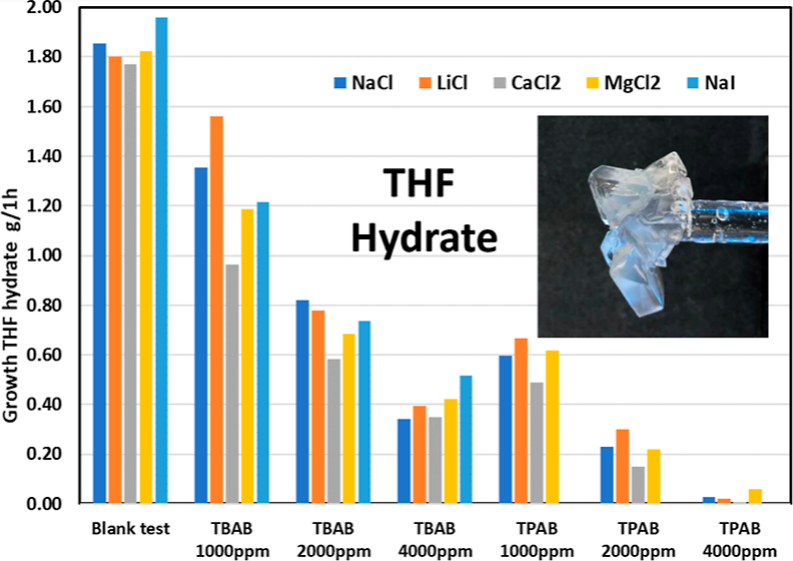

Gas hydrate anti-agglomerants (AAs) are a class of low-dosage hydrate inhibitor that are used to prevent plugging of gas hydrates in oil and condensate upstream flow lines. Industrial AAs are mostly cationic surfactants which are “hydrate-philic”, i.e., they are designed to interact with and modify gas hydrate crystal growth. Tetrahydrofuran (THF) hydrate crystal growth studies have been used for many years to determine useful functional groups to incorporate into AA surfactants. In particular, quaternary ammonium and phosphonium salts with optimized alkyl groups show good THF crystal growth inhibition, which is a key property for AAs. AAs are often screened and tested in model brines containing sodium chloride despite the produced water containing various divalent cations. Recent studies have shown that AAs performed better when tested in brines containing both sodium and calcium ions rather than just sodium ions. Here, we present THF hydrate crystal growth studies on quaternary ammonium and phosphonium salts and other related molecules including guanidinium salts and amine oxides. Tests were carried out with a variety of cations including sodium, calcium, magnesium, and lithium at identical pre-determined subcooling, in order to investigate the effect of the ion size and charge density on the crystal growth inhibition. We also investigate the effect of using the more polarizable iodide ions compared to chloride ions. Our results show that crystal growth inhibition in solutions with calcium ions is somewhat greater than that with sodium ions, in agreement with past studies on the effect of AA performance with mono- and divalent cations. However, the variation does not seem to be primarily related to the charge density and polarizing ability of the cations. This study therefore provides evidence that AAs should be tested in brines containing all the ions present in the produced water and not just sodium chloride brine.

## 1 Introduction

Gas hydrate formation and subsequent plugging of subsea flow lines are extremely difficult for operators to deal with, especially in deep water.^1−4^ Low-dosage hydrate inhibitors (LDHIs) have been developed in the last three decades to prevent gas hydrates from plugging upstream gas and oil flow lines. LDHIs come in two main classes, kinetic hydrate inhibitors (KHIs) and anti-agglomerants (AAs).^5,6^ KHIs delay the hydrate formation process, while AAs ultimately disperse any formed hydrates so that no deposits or plugs are formed. Currently, KHI formulations are limited to applications in which the subcooling (a measure of the driving force for hydrate formation in the system at a given pressure) is maximum about 8–10 °C (for a natural gas mixture) when the residence time of the fluids is high.^6−10^ This value is even lower for a methane-rich gas that instead forms structure I methane hydrate as the most thermodynamically stable phase.

While most KHI formulations contain one or more water-soluble polyamides with optimized hydrophobic groups, current AAs are predominantly cationic surfactants also with optimized hydrophobic groups.^5,6,11^ Quaternary ammonium surfactant salts are the most common. AAs can be used at much higher subcooling than KHIs as they do not need to totally prevent hydrate formation but just prevent formed hydrate particles from agglomerating and depositing problems. However, AAs are not without their limitations either. Cationic surfactants are toxic and not easily biodegraded, which limits the regions where they are still accepted for use.^6,12−14^ Due to their surfactant nature, some AAs can hamper the demulsification process, leading to a worse overboard water quality, i.e., the amount of oil in water can move above the regulated level, normally 30–40 ppm depending on the region. Improved AAs have been developed.^15^

**Figure 1:**
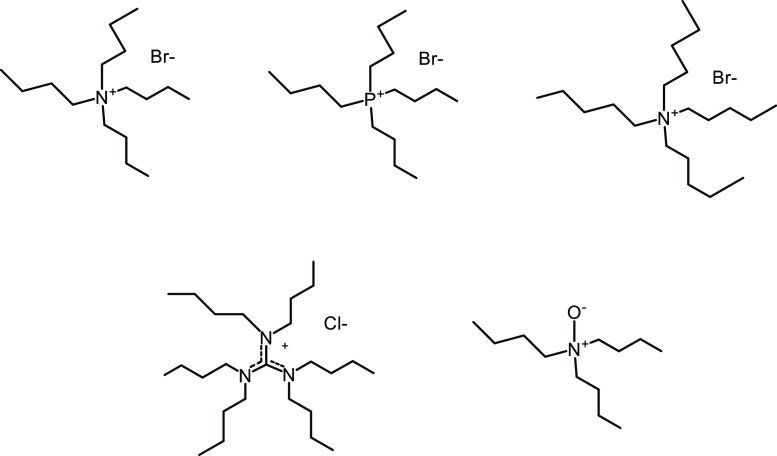
Top row, left to right, TBAB, TBPBr, and TPAB. Bottom row, left to right hexabutylguanidinium chloride (Bu6GuanCl) (left) and tributylamine oxide (TBAO).

Shell was the first to discover the anti-agglomerating behavior of quaternary ammonium and phosphonium surfactants, but this was actually based on initial studies with tetrahydrofuran (THF) hydrate.^16,17^ THF hydrate forms structure II hydrate at about 4.4 °C from the correct molar ratio mixture of THF and DI water. They found that quaternary ammonium and phosphonium salts with the correct size alkyl groups were able to slow the THF hydrate crystal growth and change the morphology. The optimum size alkyl group was n-pentyl in tetrapentylammonium bromide (TPAB), but the n-butyl group in tetrabutylammonium bromide (TBAB) and tetrabutylphosphonium bromide (TBPBr) was also very effective especially for the phosphonium salts (Figure 1). It was suggested that the quaternary salts were “hydrate-philic” in which these alkyl groups penetrate open cavities on the hydrate crystal surface impeding further growth.^6,18^ Later, it was found that end-branching of the alkyl groups (*iso*-hexyl or *tert*-heptyl) gave even better THF crystal growth inhibition.^19,20^ Other structurally related THF hydrate crystal growth modifiers were also discovered, including hexabutylguanidinium chloride (Bu6GuanCl) and tributylamine oxide (TBAO) (Figure 1).^21,22^

The first hydrate management application of quaternary ammonium salts in the oil industry was their use as synergists for KHI polymers.^23,24^ However, quaternary salts, such as TBAB or TPAB, do not inhibit SII gas hydrate nucleation when they are used alone. In fact, ammonium salts can promote hydrate formation. The reason for this may be because such salts form clathrate hydrate structures of their own, which have some of the same structural features of SII hydrates.^25,26^ This can lead to these salts being templates for the initiation of gas hydrate formation.

**Figure 2:**
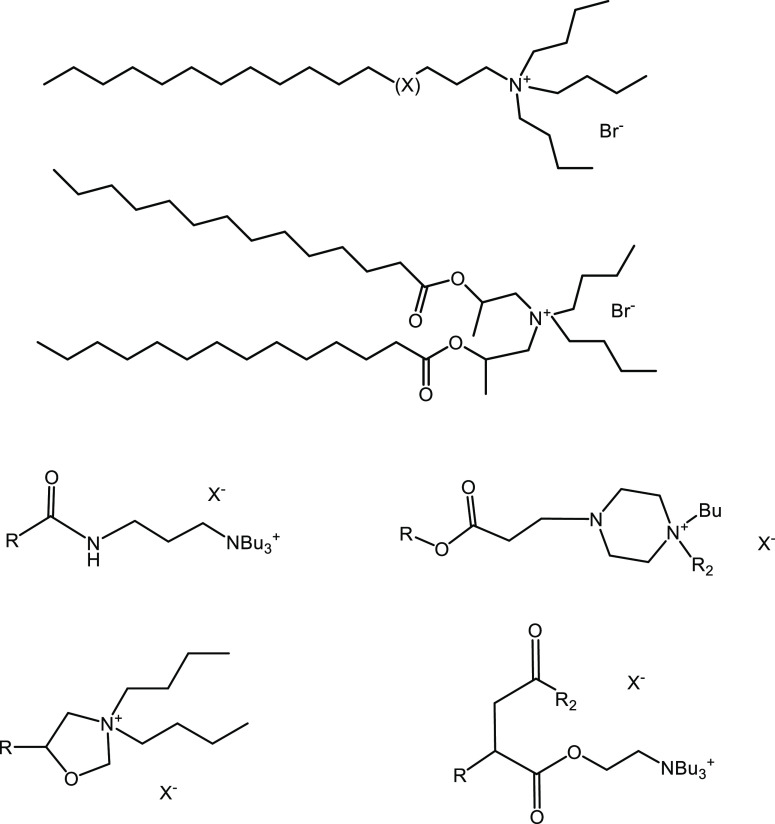
Quaternary ammonium AA surfactants, showing mono- and twin tails (top), plus other patented structures below.

The first cationic AA surfactants were made by replacing one or two n-butyl groups in TBAB with longer alkyl chains of 12–18 carbon atoms, sometimes with ester spacer groups between the chains and the nitrogen atom (Figure 2).^5,6,27,28^ Other cationic surfactants, some of which have superior properties for field applications, are also given in Figure 2. The key functional group in these AAs is still a quaternary ammonium group bonded to one or more butyl groups.

Many cationic surfactant AAs appear to work best in saline (NaCl) solution, unless the dosage is significantly increased from what is sufficient in deionized (DI) water.^5,29,30^ This is not simply an effect of lowering the subcooling by the addition of salts. Many reported AA screening studies have used 3.5–3.6% NaCl brines to model seawater.^6,11,31^ The dispersing power of some AAs improves greatly in these brines even though the subcooling is only about 1 °C less than that in DI water.^32^ However, a negative AA effect was observed using the non-ionic surfactant cocamidopropyl dimethylamine when NaCl was added.^33^

However, seawater or formation water contains other cations besides sodium, particularly divalent cations such as magnesium and calcium. A study by Servesko et al. showed that the brine composition can affect the AA performance in high-pressure rocking cell experiments. For example, brines with lower molecular weight salts needed less AA dosage.^34^ In addition, the study also showed that quaternary ammonium AAs needed somewhat lower dose in 2 wt % brine (containing both monovalent and divalent cations) than 1.5 wt % NaCl. Surprisingly, a 1:1 CsCl/LiCl blend gave a very good result with AA. In general, the study highlighted the importance to not just make the total dissolved solids based on NaCl alone when screening AAs, but to include the full produced water composition, particularly the divalent cations.

Since TBAB and the other effective quaternary ammonium and phosphonium salts were first identified as THF hydrate growth inhibitors in saline (NaCl) solutions, we wondered if switching to divalent cations in calcium or magnesium chloride would have any effect on the hydrate growth rate. York at al studied two AAs, using a model oil, water, and THF to form THF hydrates. They added either NaCl or MgCl_2_ to the mixture.^35^ Their results showed that both salts, added in sufficient quantities, could result in the agglomeration of hydrates but that MgCl_2_ led to worse agglomeration more than the dissolved NaCl. The quaternary ammonium surfactant salt used was more sensitive to dissolved salt than the nonionic rhamnolipid biosurfactant.

Chong et al. found that on top of thermodynamic inhibition, both magnesium chloride (MgCl_2_) and potassium chloride (KCl) act as kinetic inhibitors on hydrate formation, retarding the rate of formation—with KCl exhibiting a weaker inhibition compared to MgCl_2_.^36^ The weaker thermodynamic inhibitor, KCl, had a milder kinetic inhibition effect on hydrate formation and a weaker promoting effect on hydrate dissociation as compared to MgCl_2_ and sodium chloride (NaCl).

Here, we report THF hydrate crystal growth inhibition studies for quaternary ammonium salts and related molecules using THF/water solutions with various added salts. The salts include divalent cations. We comment on the relevance of these results to AA efficiency in the presence of these salts and compare the results to gas hydrate AA studies.

## 2 Experimental Methods

### 2.1 Chemicals

THF (99.9%, stabilized) was used as received from Avantor (VWR). All metal salts, TBPBr, TBAB, and TPAB, were supplied by Avantor (VWR) or Merck. Bu6GuanCl and TBAO were made by the literature methods.^21,22^

### 2.2 Test Methods

**Figure 3:**
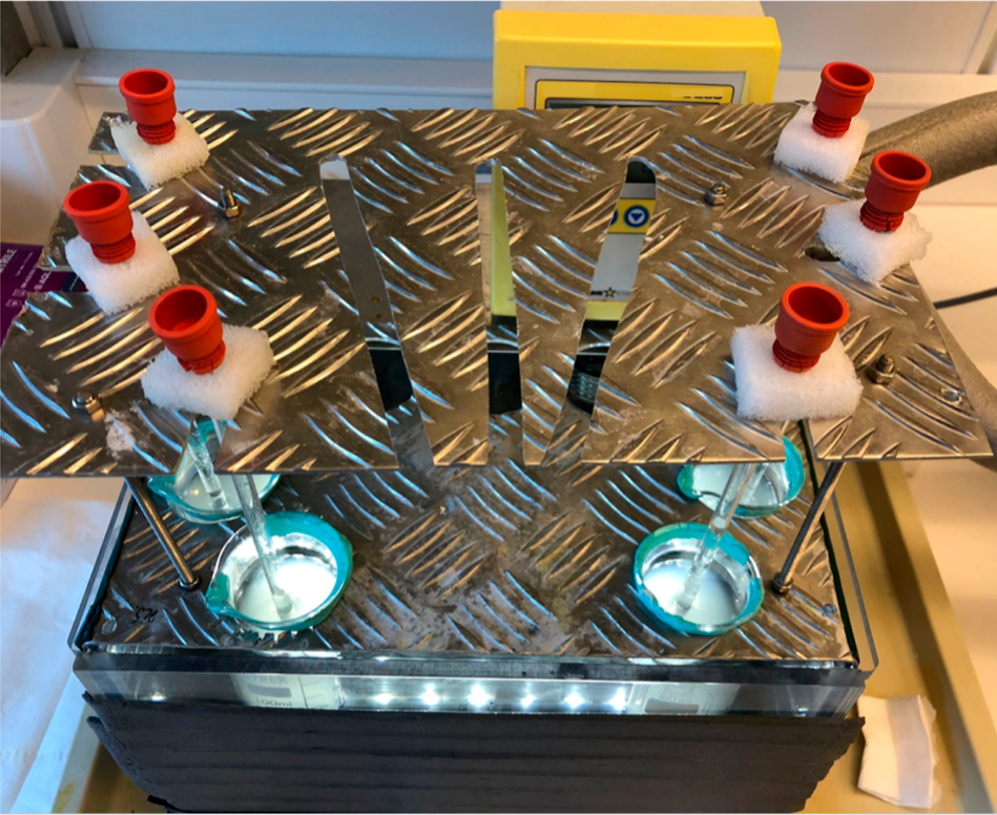
THF hydrate crystal growth equipment showing six individual tests in beakers in the cooling bath at −0.5 °C.

The standard THF hydrate crystal growth experimental method is the same as developed by Shell energy company using a mixture of H_2_O/THF/NaCl and now used by our group (Figure 3).^16,17,21,22^ In this method, NaCl (26.28 g) and THF (99.9%, 170 g) are mixed, and distilled water is added to give a final volume of 900 mL when all the salt is dissolved. This gives a stoichiometrically correct molar composition for making structure II THF hydrate, THF·17H_2_O. The salt is necessary so that the bath temperature is sub-zero to avoid melting ice in the glass tubes (see the test method below) but not so low that the THF hydrate formation rate becomes too fast. This would make it difficult to compare different growth rates if the subcooling is too high.

First, the hydrate equilibrium temperature (HET) was measured for THF mixtures with NaCl brine as well as with various salts to find the amount of salt needed to give the same HET as for NaCl brine. The HET was measured by the dissociation method described below.

#### 2.2.1 THF Hydrate Crystal Growth Test Method

The test was carried out as follows.

1. To THF (170 g) and a weighed amount of a salt (e.g., NaCl), sufficient DI water was added, and the THF and salt dissolved. More DI water was added to give 900 mL of aqueous test solution.
2. 80 mL of the aqueous test solution (water + THF + salt) was placed in each 100 mL glass beaker. Usually, six or nine beakers were run in parallel.
3. The beakers were placed in a stirred cooling bath preset to a temperature of −0.5 °C (±0.05 °C).
4. The solution was briefly stirred manually with a plastic rod every 5 min while being cooled for 20 min.
5. A hollow glass tube with inner diameter 3 mm was filled at the end with ice crystals kept at −10 °C. The ice crystals were used to initiate THF hydrate formation. The crystals are packed flat the end of the tube but do not stick out from the tube.
6. The glass tube with ice crystals was quickly placed about halfway down in the cooled THF/NaCl solution in the center of the beaker. Figure 3 shows six such tubes in glass beakers in the cooling bath.
7. THF hydrate crystals were allowed to grow at the end of the glass tube for 1 h. Some crystals may also be present in the solution.
8. The temperature in the cooling bath was then increased at approximately 2 °C/h to 3.0 °C.
9. Then, the temperature of the cooling bath is increased by 0.1 °C each hour up to 3.3 °C. This is done until all the crystals in the beaker are completely dissolved. If there are still THF hydrate crystals in the beaker, the beaker is left overnight at 3.3 °C. This was the HET for the mixture with NaCl.
10. The temperature at which all crystals melt is taken as the dissociation temperature (equilibrium temperature) of the solution.

In this project, sodium chloride (NaCl) was used first and then replaced by other salts, for example, calcium chloride (CaCl_2_·2H_2_O). The amount of calcium chloride that would give the same HET of 3.3 °C for 26.28 g of NaCl needed to be found. Therefore, the above procedure was repeated with varying amounts of calcium chloride (CaCl_2_·2H_2_O) dissolved in the same 900 mL of aqueous solution containing 170 g of THF. Once the correct amount of calcium chloride was found, this became the amount to be used in tests solutions for determining the THF hydrate growth inhibition of the quaternary ammonium salts and other additives.

#### 2.2.2 THF Hydrate Growth Inhibition Test Method

The additive to be tested was dissolved at the correct concentration (1000, 2000, or 4000 ppm) in the predetermined aqueous salt/THF solution. Steps 2–7 in the method given above for determining the HET were carried out. After 1 h, the THF hydrate crystals on the end of the glass tube were dried on a absorbent paper, cut off the tube, and weighed. This gave the THF hydrate growth rate at 1 h.

## 3 Results and Discussion

The HET for pure THF/H_2_O solution as measured by dissociation is 4.4 °C.^37^ The addition of NaCl (26.28 g in 900 mL of THF/H_2_O solution) was found to lower the HET for THF hydrate formation by about 1.1 °C from 4.4 to 3.3 °C using the method outlined in the Experimental Methods section. Next, we carried out several trials replacing NaCl with varying amounts of CaCl_2_·2H_2_O until we obtained an HET value of 3.3 °C. As with all hydrates salts in this study, the amount of water in the calcium salt was taken into account when calculating the preparation of solutions to give the same THF/water ratio. It was found that 45.5 g of calcium chloride (CaCl_2_·2H_2_O) gave HET of 3.3 °C. This amount of salt was repeated several times and with two different researchers before we were satisfied with the result. The final experiments were performed by increasing the temperature up to 3.3 °C (0.1 °C/h once 3.0 °C is reached) and letting the remaining crystals melt overnight, this experiment was carried out twice with nine samples each at a time. The next day all nine beakers were THF crystal free. It was therefore concluded that the HET was 3.3 °C when using 45.5 g of CaCl_2_·2H_2_O. The same procedure was carried out for magnesium chloride (as MgCl_2_·6H_2_O), lithium chloride (LiCl), and sodium iodide (NaI) to get the HET value as 3.3 °C. The weights and number of moles of these salts that is required are given in Table 1.

**Table 1:** Weight and Moles of Salt Required to Achieve HET of 3.3 °C for THF Hydrate^a^

salt	weight of salt/g	moles of salt or cation
NaCl	26.28	0.45
LiCl	16.5	0.39
NaI	62.0	0.41
CaCl2·2H2O	45.5	0.31
MgCl2·6H2O	54.0	0.26

a Weights given are for 900 mL of a mixed solution of salt + THF + water.

Once the correct concentrations of salt solutions that gave identical THF HET values had been determined, we were able to investigate the performance of the various additives in these solutions at the same temperature and THF hydrate subcooling. We began by comparing solutions with sodium and calcium ions as calcium is usually the divalent cation of highest concentration in oilfield formation water. It should be noted that in order to get identical HET values, the concentration of the cations in the THF/H_2_O/salt solutions is not the same.

**Figure 4:**
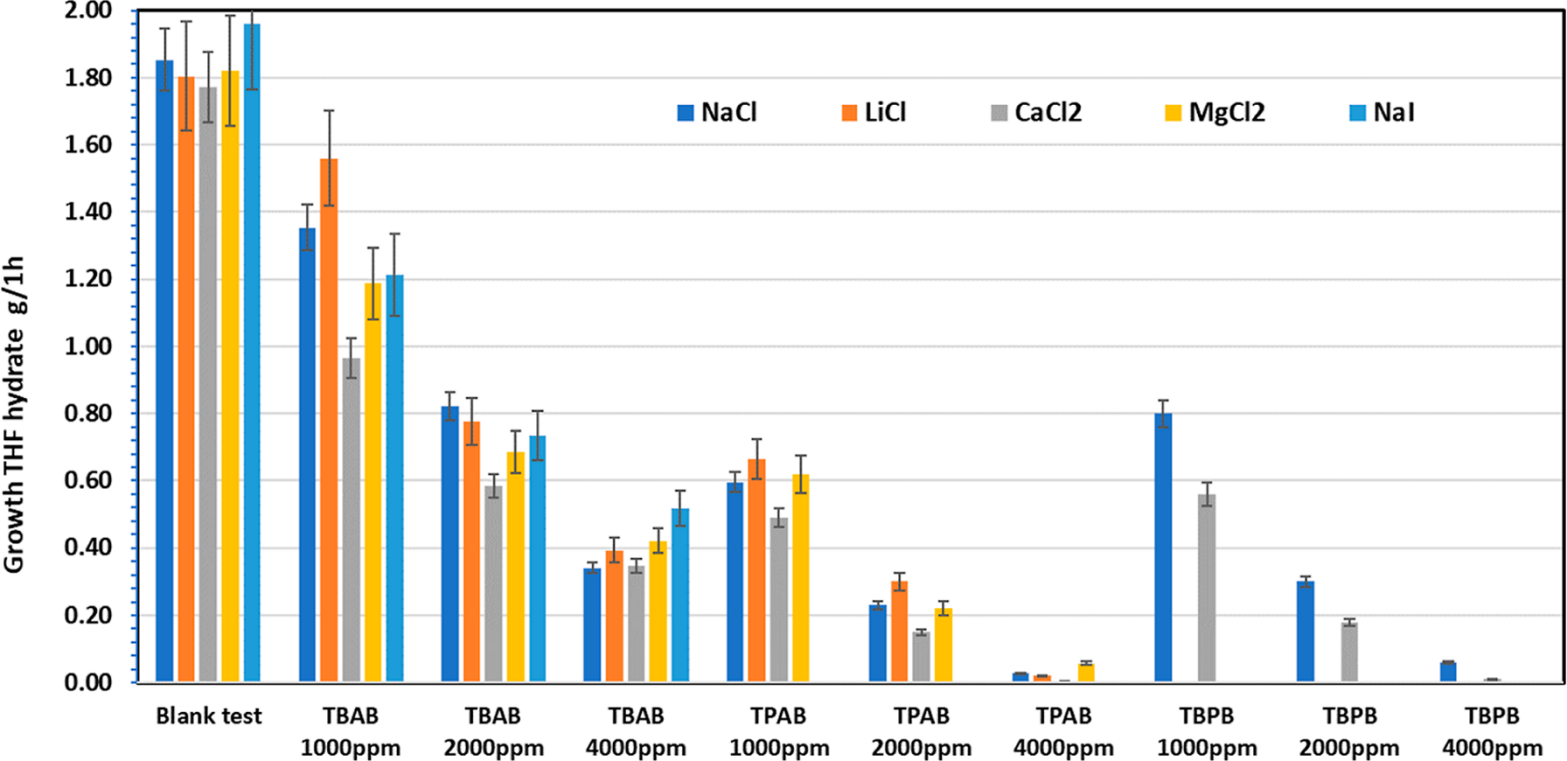
THF hydrate growth in 1 h for the three quaternary onium salts in various salt solutions.

**Figure 5:**
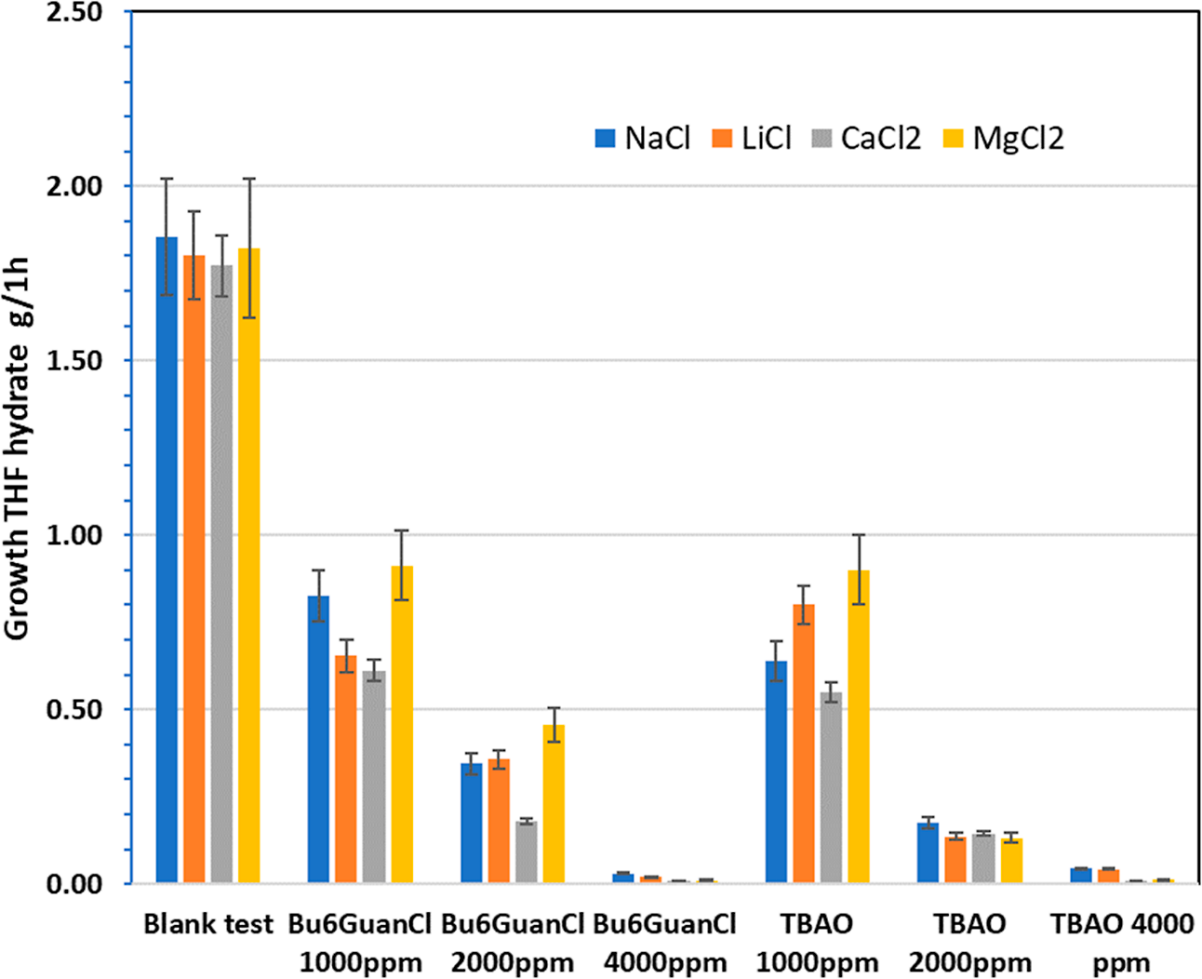
THF hydrate growth in 1 h for TBAO and Bu6GuanCl in various salt solutions.

Table 2 lists the THF hydrate crystal growth results in sodium and calcium brines for blank tests and the five additives investigated, TBAB, TPAB, TBPB, TBAO, and Bu_6_GuanCl. The same results can also be found graphically in Figures 4 and 5 together with results with other salts. All growth rates are given as the average of 9–10 individual tests. The standard deviation in growth rates is about ± 10–12% if tests are performed carefully. The test has some difficulties. If the ice falls out of the tube into the aqueous THF/salt solution, the test is ruined. If the ice protrudes out of the tube too far, it gives rise to too much THF hydrate growth. Conversely, if the ice is not at the very end of the tube, sometimes covered by an air bubble or insoluble test material, the THF hydrate growth is lower than expected. Each tube must be carefully examined after placing in the center of the beaker to make sure the result will be reliable. When removing the tube, it is important to weigh only the crystals growing from the end of the tube, initiated by the ice in the tube.

**Table 2:** THF Hydrate Growth in 1 h with Various Additives Using Sodium and Calcium Ions

additive	additive concentration/ppm	weight of THF hydrate/g	
		Na^+^	Ca^2+^
no additive		1.85	1.77
TBAB	1000	1.34	0.97
	2000	0.82	0.58
	4000	0.34	0.35
TPAB	1000	0.60	0.49
	2000	0.23	0.14
	4000	0.03	0.01
TBPB	1000	0.80	0.56
	2000	0.30	0.18
	4000	0.06	0.00
TBAO	1000	0.64	0.55
	2000	0.34	0.16
	4000	0.01	0.01
Bu_6_GuanCl	1000	0.83	0.61
	2000	0.34	0.18
	4000	0.03	0.01

**Figure 6:**
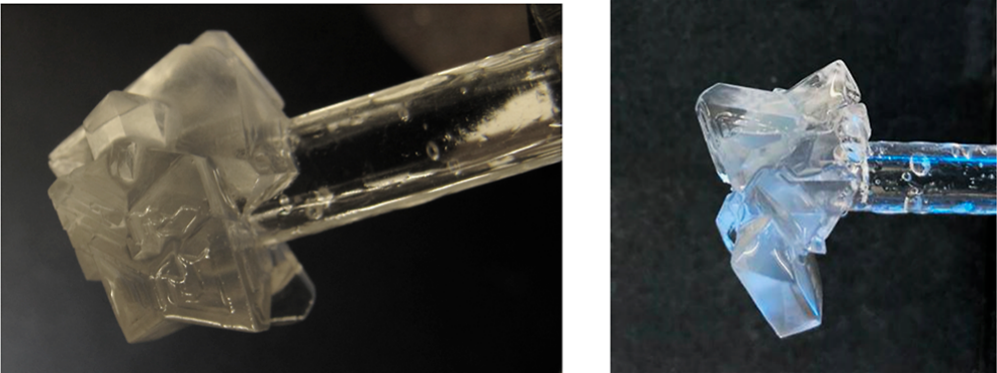
THF hydrate crystals after 1 h growth at −0.5 °C. Left: no additive in aq. NaCl/THF. Right: no additive in aq. NaI/THF.

**Figure 7:**
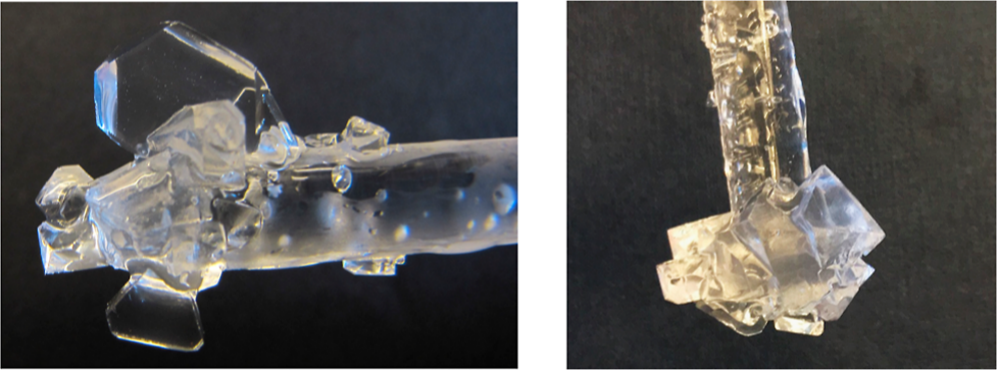
THF hydrate crystals after 1 h growth at −0.5 °C. Left: 2000 ppm TBAO in aq. CaCl_2_/THF. Right: 1000 ppm TBAB in aq. NaCl/THF.

Examples of THF hydrate crystals on the glass tube are shown in Figures 6 and 7. With no additive or a low concentration of additive (1000 ppm), we observed pyramidal crystals or sometimes thick plates. The five additives chosen for this study are all good THF hydrate crystal growth inhibitors. Therefore, at high concentrations (2000–4000 ppm), the regular structure of the THF hydrate crystals was modified to give more rounded edges, as Shell first observed with the quaternary ammonium and phosphonium salts.

For the blank tests with no additive, the average THF crystal growth in 1 h was 1.85 g for sodium brine and 1.77 g for calcium brine. These results are not significantly different. However, we observed a significantly lower growth rate for calcium brine than that for sodium brine for all five additives at most concentrations. In addition, the growth rate of THF hydrate decreases with increasing additive concentration in both calcium or sodium brines. The effect of calcium is more striking given that the molar concentration of calcium ions is about 31% lower than that for sodium ions [100 × (0.45 – 0.31/0.45) = 31%].

This increased inhibitory effect of calcium over sodium is in agreement with the AA results of Servesko et al., in which brines containing calcium ions gave better AA effect than brines with just sodium ions.^34^ The head groups in commercial AAs are often quaternary ammonium or phosphonium bonded to one or more butyl groups. Thus, the THF hydrate results help explain the improved effect of AAs in brines containing calcium as the calcium appear as to help slow hydrate crystal growth, a key step in the “hydrate-philic” AA mechanism.^38^ Four of the five additives investigated have active cationic groups, quaternary ammonium, phosphonium, and guanidinium. However, the positive effect of calcium ions compared to sodium ions was also observed for the neutral molecule TBAO as well. Therefore, the charge on the additive does not seem to be significant in order to see the positive effect of the calcium ions.

The trend in the effect on THF hydrate growth for magnesium ions, the second most prevalent divalent cation in oilfield formation water, was not as clear as observed for calcium ions (Figures 4 and 5). The blank test without additive and most tests with additives showed no significant improvement in the THF hydrate growth rate for solutions with magnesium ions compared to sodium ions. In fact, for some tests, we observed slightly higher growth rates with magnesium ions.

How can these observations be explained? Metal cations such as magnesium and calcium ions will bind to THF as a ligand to form complexes.^39^ This is probably taking place in the aqueous THF/salt solutions but is irrelevant for gas hydrate AAs where no THF is present. A possible solution is the relative charge density of the cations and their ability to hydrate water. The charge density relates to the polarizing ability of a metal cation according to Fajan’s rules.^40^ The more polarizing a cation is, the stronger the hydration in aqueous solution, or the stronger the binding to THF via the ring oxygen atom. Table 3 lists the polarizing ability of selected cations and chloride ion compared to the THF hydrate growth rates obtained. These values vary somewhat in the literature depending on the source of the data, but the ranking is always the same. We have labeled the growth rates roughly as lowest, middle, and highest. Magnesium ions are known to have higher polarizing as a function of its small size compared to calcium, while sodium ions have lower polarizing ability than either divalent cation. Yet, the THF growth rates are similar for sodium and magnesium ion solutions but worse than calcium ion solutions. Therefore, we can conclude that the charge density on the metal cation does not correlate with THF hydrate inhibition ability, with or without additives.

**Table 3:** Polarizing Power of the Investigated Cations Compared to Results for the THF Hydrate Growth Rate

cation	charge densities	polarizing power	THF hydrate growth rating
Na^+^	24	1.0	middle
Li^+^	52	1.7	highest
Ca^2+^	52	2.0	lowest
Mg^2+^	120	3.9	middle

This conclusion is further backed up by tests with lithium chloride (LiCl). Li^+^ ions are more polarizing than Na^+^ ions (Table 3). The blank LiCl/THF solution gave similar growth rate as a NaCl/THF solution. Four of the five additives were tested using aqueous LiCl/THF solution. Only at low concentrations of TBAB, there was a small decrease in the THF hydrate growth rate compared to the NaCl/THF solution and even then, this was borderline significant. We carried out *t* tests on the 10 experiments for each salt solution, and the *p*-value was 0.05 giving only 95% confidence that the difference in the results is significant.^41^

Given that the charge density of the cations does not correlate with the THF hydrate growth rates, we wondered if the size of the cation was more relevant. Ca^2+^ was the largest cation investigated. It is possible that this divalent cation has more voluminous perturbation of the bulk water structure than smaller ions, such as Na^+^ or even the divalent Mg^2+^. The difference in hydration shells of magnesium and calcium is not straightforward, with varying modeling results reported.^42,43^ Analysis of the hydrogen-bonded structure of water in the vicinity of calcium ions shows that the average number of hydrogen bonds per water molecules, which is 1.8 in pure liquid water, decreases as the concentration of alkali-halide salts in solution increases.^44^ Other cations were mot modeled in this study. Another modeling study suggested that the global minimum for the hydration shell of Mg^2+^ is represented by a quite stable octahedral arrangement of six coordinated water molecules, whereas for Ca^2+^, the hydration structure is highly variable.^45^

Another speculation is that the larger Ca^2+^ ions interact with water molecules on the hydrate surface better than smaller ions like Na^+^ or Mg^2+^, which could arrest the hydrate growth. In this way, the cation acts like a weak synergist with the additives in the THF hydrate growth test or indeed the AA in gas hydrate tests. We plan to investigate larger cations such as Sr^2+^ or divalent anions such as SO_4_^2–^ to explore this further.

We did carry out some tests with sodium iodide as a comparison of a more polarizable anion than chloride in sodium chloride. Tests were carried out only for the blank solution with no additive and TBAB as a model quaternary ammonium salt (Figure 4). TBAO and Bu6GuanCl were found to be only partially soluble in the aqueous NaI/THF solution at 1000–4000 ppm and were therefore not investigated for the THF hydrate growth rate. The blank NaI/THF aqueous solution gave a growth rate that was not significantly different to NaCl/THF. The same was also true of TBAB at 1000 and 2000 ppm. At 4000 ppm TBAB, the growth rate was significantly higher in the NaI/THF solution, over 50% of the value in NaCl/THF (0.52 g vs 0.34 g/h, respectively). A possible reason for this is ion pairing between iodide ions and the tetrabutylammonium cation. The ion pairing is expected to be stronger than that with bromide ions (in TBAB) or chloride ions (in NaCl) as the iodide ions are more polarizable and will have most effect on the growth rate at the highest TBAB concentration.

## 4 Conclusions

The concentrations of various salts added to THF/water solutions that give the same THF HET were determined. THF hydrate crystal growth experiments over 1 h were conducted for these aqueous salt/THF solutions at −0.5 °C. For blank solutions without additives, there was no significant difference observed in the amount of THF hydrate growth between the various salts.

Five additives known to have a strong inhibitory effect on THF hydrate crystal growth were investigated in the various aqueous salt/THF solutions. They were three quaternary onium salts (TBAB, TPAB, and TBPB), a guanidinium salt (Bu6GuanCl), and an amine oxide (TBAO). Only in the CaCl_2_/THF solution did we observe a significant reduced rate of THF hydrate growth compared to the standard NaCl/solution. The effect is not very large but agrees well with published results on “hydrate-philic” cationic surfactant AA tests that performed better in brines containing calcium and not just sodium ions.

The results indicate that the effect of the cations on the rate of hydrate crystal growth is an important part of the AA mechanism for the quaternary surfactants. It also underlines the importance for operators and service companies to evaluate the performance of AAs with all the cations present in the produced water at the correct concentration and not just use a sodium chloride brine. This is especially true for calcium ions. The reason for the enhanced inhibition afforded by the calcium ions is not fully understood. The charge density and polarizing power of this cation do not appear to be critical given that the more polarizing divalent magnesium ion does not lead to better inhibition of THF hydrate growth than calcium or sodium ions.
